# Analysis of the association between different domains and intensities of physical activity in adults: An observational and cross-sectional study

**DOI:** 10.1371/journal.pone.0306873

**Published:** 2024-10-10

**Authors:** Diego G. D. Christofaro, William R. Tebar, Gerson Ferrari, Amanda B. dos Santos, Jeffer E. Sasaki, Raphael M. Ritti-Dias, Gabriel G. Cucato

**Affiliations:** 1 Department of Physical Education, School of Technology and Sciences, São Paulo State University (Unesp), Presidente Prudente, SP, Brazil; 2 Faculty of Health Sciences, Universidad Autónoma de Chile, Providencia, Chile; 3 Departament of Physical Education, Universidade Federal do Triângulo Mineiro (UFTM), Uberaba, MG, Brazil; 4 Universidade Nove de Julho–Postgraduate program in Rehabilitation Sciences, São Paulo, SP, Brazil; 5 Department of Sport, Exercise and Rehabilitation, Northumbria University, Newcastle upon Tyne, United Kingdom; University of Tartu, ESTONIA

## Abstract

**Background and objective:**

To analyze the association between different domains and intensities of physical activity (PA) in adults.

**Methods:**

269 participants were randomly selected in a community-dwelling representative sampling process. The habitual PA practice was assessed in different domains (occupational, sports practice, and leisure time/commuting) using Baecke’s questionnaire and in different intensities (light, moderate, vigorous, and very vigorous) by accelerometry. Linear regression models analyzed the association between variables.

**Results:**

Moderate and moderate-to-vigorous PA was positively associated with the three PA domain scores. Vigorous PA was associated with sports practice and leisure time/commuting PA. Only sports practice was associated with very vigorous-intensity PA (β = 1.32; 95% CI = 0.29; 2.35). Different associations were observed in stratified analyses according to sex and age groups. Men and younger participants showed an association of domain scores with higher intensity PA than their counterparts.

**Conclusion:**

The findings suggest that higher intensities of PA were observed primarily in leisure time/commuting PA and sports practice, and this association varied according to sex and age.

## Introduction

Habitual physical activity (PA) has been associated with several long-term health benefits, such as a lower risk of cardiovascular disease, musculoskeletal pain, and psychological disorders, such as anxiety and depression, among other benefits in diverse populations [1, 2]. PA has been considered an important tool for improving the general health of community-living people, especially when engaging in high-intensity activities [3]. Lack of physical activity or physical inactivity can harm the healthcare system economically. In a systematic review, Ding et al. [4] observed that physical inactivity costs 53.8 billion dollars for health systems worldwide.

Recent recommendations for PA suggest that adults should engage in 150–300 minutes of moderate-intensity or 75–150 minutes of vigorous-intensity weekly [5]. The recommendation also stimulates engaging in various PA domains such as occupational activities (e.g., domestic services and/or work), leisure time (e.g., sports, weight training, walking, dancing, among others), and active commuting (e.g., walking, bicycle, or other form of active transport to work, school, or shopping).

Considering the different intensities of PA, some studies have shown that very vigorous and/or vigorous PA tends to be performed during sports [6, 7], which can mainly provide cardiovascular benefits and bone health [8, 9]. On the other hand, moderate PA can be developed through commuting PA [10], while light PA tends to be developed through domestic tasks, casual walking, and other activities of daily living [11]. These activities provide health benefits to numerous populations [11–13].

In this sense, PA has been measured in subjective and objective ways. The most used instrument to measure PA subjectively is questionnaires. Among the best-known questionnaires, we can mention the International Physical Activity Questionnaire (IPAQ) [14] and a short questionnaire for the measurement of habitual physical activity in epidemiological studies developed by Baecke et al. [15]. Regarding the direct measurement of PA, mention may be made of doubly marked water (an extremely expensive method and difficult to use on a large scale) and accelerometry (a device that generally measures PA in three axes: vertical, horizontal anteroposterior and medio-lateral). The advantage of measuring PA through questionnaires is that it is possible to analyze different domains (however, intensity information is hampered by memory bias). In contrast, direct measurement using accelerometry provides precise intensity information (although it is not possible to identify the PA domain).

However, most studies have separately presented the benefits of different intensities and/or health-related domains. This is one of the first studies carried out in a Latin American country to estimate which PA domains and different intensities are more likely to be carried out. Another positive point in the present study’s analysis is that we considered the differences between sex and age, variables that can influence the practice of PA.

Thus, the present study aimed to assess the relationship between different intensities and domains of PA in a randomly selected sample of community-dwelling adults.

## Methods

### Sample and Ethical procedures

**Fig 1:**
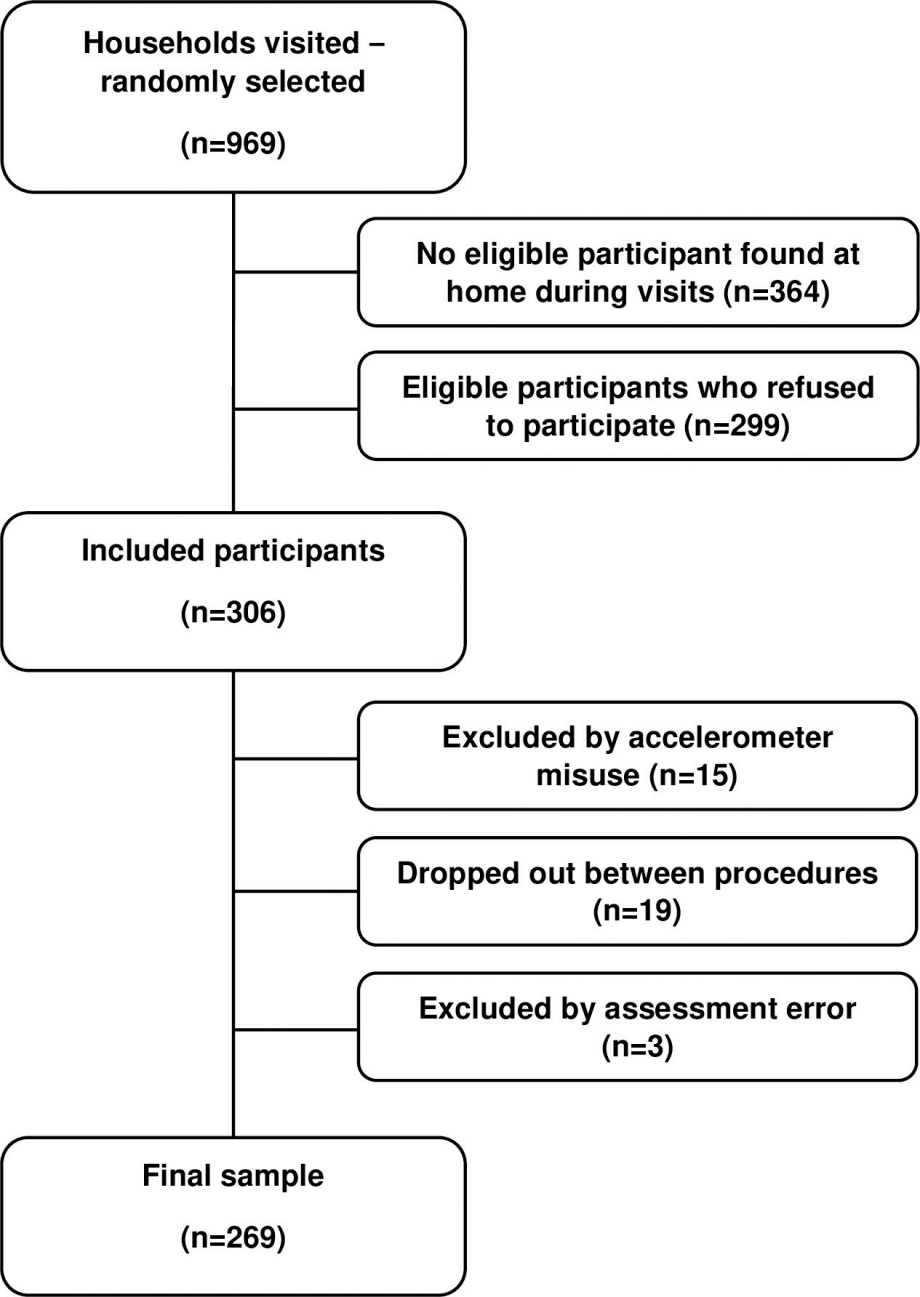
Sampling flowchart.

This is a cross-sectional study. The sample consisted of adults aged 18 years or over living in a small city in the southeastern region of Brazil with approximately 20 thousand inhabitants. For the selection of the sample, the participants were randomized considering the census sector (n = 23), the neighborhood, the street, the blocks, and the house. In each one of the census tracts, the proportionality of residents compared to the overall population was considered to reach an equal proportion in the study sample [16]. The following inclusion criteria were used: (i) being 18 years old years or more; (ii) being free of physical limitations or disabilities that precluded the use of an accelerometer in a standing position; (iii) having signed the Free and Informed Consent Form. Participants were excluded according to the following criteria: (i) misuse of the accelerometer; (ii) dropout between research procedures; (iii) absence of data for any included variable [17]. The sampling flowchart is presented in **Fig 1**.

All procedures were approved by the Ethics and Research Committee of São Paulo State University (CAAE: 72191717.9.0000.5402). All participants signed a consent form agreeing to participate in the study. Participant recruitment began on August 1, 2018, and ended on November 31, 2019.

### Domains of physical activity

The PA domains were analyzed using the questionnaire by Baecke et al. [15]. This questionnaire is divided into three domains that consider the PA developed in occupational activities, sports practice, and leisure time/commuting and is validated for the Brazilian population [18]. In occupational PA, domestic tasks were considered, as to perform these tasks, it is necessary to walk and carry loads, squat, and stand, among other tasks, which can promote fatigue. The second section comprises four questions regarding the sports practice at leisure, the number of hours, intensity, and frequency of physical exercise performed during the week, and the time (in months) that PA has been practiced. The third section contains four questions about leisure time and commuting PA, which refer to the frequency of watching TV, walking and cycling and daily time spent in active commuting. All participants of this study were interviewed face-to-face by researchers.

### Intensities of physical activity

PA intensity was assessed using the ActiGraph GT3X accelerometer (ActiGraph, LLC, Pensacola, FL, USA). Participants were instructed to wear the accelerometer laterally at the waist for seven days during waking hours.

Once the accelerometer was returned, data were downloaded to a computer using the ActiLife software. Raw acceleration data were subsequently reintegrated to 60s epoch data. To determine wear time, we applied the automated Troiano Algorithm. At least 10 hours of daily use was required to consider a day valid. The minimum number of valid days necessary to include the participant’s data in the analysis was five days (4 weekdays and 1 weekend day).

The cutoff points used were those developed by Aguilar-Farías et al. [19] and Sasaki et al. [20], in which activities with less than 200 counts per minute were considered sedentary (Aguilar-Farías et al. 2014), 201–2690 counts per minute as light, 2691–6166 counts per minute as moderate, 6167–9642 as vigorous and ≥9643 as very vigorous PA [20]. The weekly minutes were computed for data analysis according to each PA intensity into light, moderate, moderate-to-vigorous (MVPA), vigorous, and very vigorous.

### Socioeconomic level

The Brazilian Criteria for Economic Classification was used to assess socioeconomic level [21], which was considered as a covariate. The tool assesses consumer goods, household amenities, and the participant’s education level to determine their socioeconomic status. It uses a classification system from A to E, where A represents the highest level and E the lowest.

### Statistical analysis

The sample characterization variables were presented as means and standard deviations. Continuous variables were compared according to sex using an independent t-test for averaged variables and a skewed distribution using the U Mann-Whitney test. Linear regression models were used to analyze the association between the scores of physical activity in specific domains (occupational, sports practice, and leisure time/commuting) with the time spent in different intensities of physical activity (light, moderate, vigorous, and very vigorous), adjusted by sex, age, and socioeconomic level. Stratified analysis was performed according to sex and age group (less than 40 years, between 40–59 years, and 60 years and more). The analysis was conducted using SPSS 25.0 statistical software, and a significance level of p<0.05 with a 95% confidence interval was considered.

## Results

The sample consisted of 269 participants (154 women, 57.2%) with an average age of 42.4 ±17.0 years (minimum 18.0 and maximum 84.0 years old, with a skewed distribution). Occupational PA was the domain with the highest score of PA, whereas light-intensity PA was the most practiced PA in the sample. The characteristics of the sample are presented in **Table 1**.

**Table 1:** Sample characterization.

Variables	Mean (SD)		
	Overall sample (n = 269)	Women (n = 154)	Men (n = 115)
Age (years)	42.4 (17.0)	44.4 (17.2)	39.8 (16.6)*
Weight (kg)	77.0 (15.8)	72.0 (13.8)	83.6 (15.9)*
Height (cm)	165.6 (9.7)	159.5 (6.9)	173.6 (6.7)*
Body mass index (kg/cm^2^)	28.1 (5.2)	28.4 (5.1)	27.8 (5.4)
**PA domains**			
Occupational PA (Baecke score)	2.8 (1.0)	2.9 (1.1)	2.8 (1.0)
Sports practice (Baecke score)	2.4 (0.8)	2.4 (0.8)	2.6 (0.9)*
Leisure time/commuting PA (Baecke score)	2.3 (0.7)	2.2 (0.7)	2.5 (0.8)*
**PA Intensity**			
Light (min/week)	2530.5 (765.6)	2558.5 (772.5)	2489.4 (762.3)
Moderate (min/week)	185.1 (129.5)	170.1 (118.3)	205.4 (142.9)*
MVPA (min/week)	199.0 (138.2)	179.6 (125.9)	224.6 (151.4)*
Vigorous (min/week)	12.4 (23.2)	8.8 (21.6)	16.7 (23.8)*
Very vigorous (min/week)	1.4 (6.8)	0.7 (2.2)	2.5 (10.1)*

PA = Physical activity; MVPA = Moderate to vigorous physical activity.

**Table 2** shows the association between the different domains and intensities of PA among the entire sample. Occupational score was related to light (β = 198.43 [95% CI = 107.41; 289.45]), moderate (β = 24.26 [95% CI = 9.59; 38.53]) and MVPA intensity (β = 24.13 [95% CI = 8.55; 39.70]). Sports practice score was associated with moderate (β = 25.29 [95% CI = 5.63; 44.95]), MVPA (β = 32.91 [95% CI = 12.22; 53.60), vigorous (β = 6.30 [95% CI = 2.95; 9.46]) and very vigorous intensity (β = 1.32 [95% CI = 0.29; 2.35]). Leisure time/Commuting PA score was associated with light (β = 141.78 [95% CI = 7.85; 275.58), moderate (β = 32.57 [95% CI = 11.12; 54.02]), MVPA (β = 36.77 [95% CI = 14.10; 59.43]) and vigorous intensity (β = 4.03 [95% CI = 0.30; 7.76]).

**Table 2:** Association between different domains and intensity of physical activity in adults (n = 269).

	Light PA	Moderate PA	MVPA	Vigorous PA	Very vigorous PA
	β(95%CI)	β(95%CI)	β(95%CI)	β(95%CI)	β(95%CI)
**Occupational PA**	**198.43 (107.41; 289.45)**	**24.26 (9.59; 38.53)**	**24.13 (8.55; 39.70)**	-0.22 (-2.80; 2.35)	0.09 (-0.69; 0.87)
**Sports practice**	101.04 (-19.78; 221.68)	**25.29 (5.63; 44.95)**	**32.91 (12.22; 53.60)**	**6.30 (2.95; 9.46)**	**1.32 (0.29; 2.35)**
**Leisure time/ Commuting PA**	**141.78 (7.85; 275.58)**	**32.57 (11.12; 54.02)**	**36.77 (14.10; 59.43)**	**4.03 (0.30;7.76)**	0.16 (-0.98; 1.30)

PA = Physical activity; MVPA = Moderate-to-vigorous physical activity; CI = Confidence interval. Analysis adjusted by sex, age and socioeconomic status. Bold values were statistically significant at p<0.05 level.

The association between different domain scores and the intensity of PA according to sex is presented in **Table 3**. Among women, the association of sports practice scores with vigorous and very vigorous PA was no longer significant, as observed in the entire sample. In contrast, all the other previous associations remained. Regarding male participants, the sports practice score (β = 8.21 [95% CI = 3.13; 13.28]) and leisure-time/commuting score (β = 7.05 [95% CI = 1.38; 12.72]) were associated with vigorous PA only, while occupational score remained associated with light (β = 154.40 [95% CI = 7.39; 301.40]) and moderate-intensity PA (β = 28.41 [95% CI = 2.37; 54.44]).

**Table 3:** Association between different domains and intensity of physical activity in adults, according to sex (n = 269).

	Light PA	Moderate PA	MVPA	Vigorous PA	Very vigorous PA
	β(95%CI)	β(95%CI)	β(95%CI)	β(95%CI)	β(95%CI)
**Women**					
Occupational PA	**214.36 (97.09; 331.64)**	**22.84 (5.63; 40.05)**	**23.70 (5.31; 42.08)**	0.89 (-2.38; 4.15)	-0.03 (-0.36; 0.30)
Sports practice	-0.40 (-168.98; 168.17)	**30.64 (5.91; 55.38)**	**35.18 (8.88; 61.48)**	4.24 (-0.40; 8.87)	0.30 (-0.17; 0.77)
Leisure time/ Commuting PA	**229.98 (53.66; 406.31)**	**55.30 (29.86; 80.74)**	**56.56 (29.28; 83.82)**	1.16 (-3.85; 6.17)	0.10 (-0.40; 0.60)
**Men**					
Occupational PA	**154.40 (7.39; 301.40)**	**28.41 (2.37; 54.44)**	26.52 (-0.99; 54.04)	-2.03 (-6.28; 2.22)	0.15 (-1.70; 2.00)
Sports practice	173.03 (-5.81; 351.86)	22.33 (-10.48; 55.13)	32.65 (-1.59; 66.90)	**8.21 (3.13; 13.28)**	2.12 (-0.15; 4.38)
Leisure time/ Commuting PA	10.55 (-195.42; 216.51)	6.66 (-29.61; 42.92)	13.74 (-24.36; 51.83)	**7.05 (1.38; 12.72)**	0.03 (-2.49; 2.55)

PA = Physical activity; MVPA = Moderate-to-vigorous physical activity; CI = Confidence interval. Analysis adjusted by age and socioeconomic status. Bold values were statistically significant at p<0.05 level.

**Table 4** presents the association of PA domains with different intensities according to age group. Among participants with less than 40 years old, occupational score was associated with light-intensity PA (β = 247.90 [95% CI = 116.02; 379.79]), sports practice score was associated with vigorous (β = 8.38 [95% CI = 3.42; 13.34]) and very vigorous PA (β = 2.55 [95% CI = 0.43; 4.68]), and leisure time/commuting score was associated with vigorous-intensity PA (β = 6.37 [95% CI = 0.49; 12.25]). When considering the participants between 40–59 years old, the three domain scores were associated with moderate PA (occupational β = 34.46 [95% CI = 3.37; 65.55]; sports practice β = 45.56 [95% CI = 4.59; 86.52]; and leisure time/commuting β = 45.66 [95% CI = 5.89; 85.44]) and MVPA (occupational β = 35.51 [95% CI = 2.22; 68.81]; sports practice β = 51.91 [95% CI = 8.27; 95.54]; and leisure time/commuting β = 48.95 [95% CI = 6.44; 91.46]). Regarding older participants (60 years old and more), sports practice and leisure time/commuting scores were associated with moderate PA (β = 57.72 [95% CI = 14.34; 101.11] and β = 69.73 [95% CI = 17.03; 122.43], respectively) and MVPA (β = 59.04 [95% CI = 15.11; 102.98] and β = 70.29 [95% CI = 16.78; 123.80], respectively), whereas occupational PA was no longer associated with PA intensity as in younger groups.

**Table 4:** Association between different domains and intensity of physical activity in adults, according to age group (n = 269).

	Light PA	Moderate PA	MVPA	Vigorous PA	Very vigorous PA
	β(95%CI)	β(95%CI)	β(95%CI)	β(95%CI)	β(95%CI)
**Less than 40 years-old**					
Occupational PA	**247.90 (116.02; 379.79)**	4.06 (-16.24; 24.36)	1.40 (-20.48; 23.28)	-2.92 (-7.30; 1.45)	0.26 (-1.58; 2.11)
Sports practice	102.55 (-55.17; 260.27)	7.18 (-16.71; 31.06)	18.11 (-7.45; 43.67)	**8.38 (3.42; 13.34)**	**2.55 (0.43; 4.68)**
Leisure time/ Commuting PA	8.70 (-178.43; 195.84)	8.92 (-18.64; 36.48)	15.83 (-13.77; 45.43)	**6.37 (0.49; 12.25)**	0.54 (-1.97; 3.05)
**Between 40 and 59 years-old**					
Occupational PA	59.71 (-112.03; 231.45)	**34.46 (3.37; 65.55)**	**35.51 (2.22; 68.81)**	1.02 (-4.62; 6.66)	0.03 (-0.27; 0.33)
Sports practice	119.91 (-98.73; 338.56)	**45.56 (4.59; 86.52)**	**51.91 (8.27; 95.54)**	6.44 (-0.88; 13.75)	-0.08 (-0.48; 0.31)
Leisure time/ Commuting PA	119.66 (-93.63; 332.95)	**45.66 (5.89; 85.44)**	**48.95 (6.44; 91.46)**	3.35 (-3.85; 10.55)	-0.06 (-0.45; 0.32)
**60 years-old and more**					
Occupational PA	148.43 (-96.94; 393.80)	12.57 (-21.43; 46.56)	13.25 (-21.22; 47.71)	0.76 (-0.88; 2.40)	-0.08 (-0.20; 0.04)
Sports practice	-34.19 (-358.56; 290.18)	**57.72 (14.34; 101.11)**	**59.04 (15.11; 102.98)**	1.26 (-0.98; 3.50)	0.06 (-0.11; 0.23)
Leisure time/ Commuting PA	330.46 (-58.32; 719.25)	**69.73 (17.03; 122.43)**	**70.29 (16.78; 123.80)**	0.54 (-2.22; 3.30)	0.02 (-0.18; 0.23)

PA = Physical activity; MVPA = Moderate-to-vigorous physical activity; CI = Confidence interval. Analysis adjusted by sex and socioeconomic status. Bold values were statistically significant at p<0.05 level.

## Discussion

The present study aimed to analyze the relationship between the different intensities and domains of PA in a representative sample of community-dwelling adults. Light-intensity PA was associated with occupational and commuting PA. Moderate-intensity and MVPA were positively related to occupational, sports practice, and leisure time/commuting PA. Vigorous-intensity PA was associated with sports practice and leisure time/commuting PA. Finally, only sports practice was associated with very vigorous-intensity PA.

Light intensity was associated with occupational and commuting PA. Quinn et al. [22] showed that light PA can vary according to the type of occupational PA. MVPA was the only type of intensity associated with the three different domains analyzed in this study. Several health organizations recommend moderate-intensity PA as an essential lifestyle habit for preventing and treating chronic diseases [5]. This study observed that the occupational PA domain was associated with moderate-intensity PA. This relationship may have occurred due to the sample’s characteristics. The city where this study was carried out has approximately 20 thousand inhabitants, and about 55% of the sample was composed of blue-collar workers (butchers, cleaning assistants, firefighters, and domestic workers). Previous studies have shown that work demands can have a relative physical effort [22, 23]. Yamamoto et al. [24] observed that more active participants in occupational activities had a lower risk of developing chronic diseases. In the analysis stratified by age group, the present study observed that occupational PA was associated with light-intensity PA among younger participants and moderate intensity among older age groups. This association can be related to younger people’s higher use of technological devices [25], which may also be reflected in occupational tasks requiring minimum physical effort.

Sports practice was the only domain associated with higher-intensity physical activity in the study. Sports practice is a free-choice and planned behavior that favors the constant adoption of higher intensities of PA, which may improve the health status and physical fitness of other PA domains [26]. According to sex, the present study observed that sports practice was associated with moderate-intensity PA among women and vigorous-intensity PA among men. This finding aligns with previous studies that reported that men achieve higher absolute performance than women in sports activities [27–29]. The present study observed similar associations when stratified by age group, where sports practice was associated with vigorous-intensity PA in the younger group and moderate-intensity PA in older groups. Kujala et al. [28] also reported higher PA intensity in younger participants compared to older participants, and this association can be substantially related to the decrease in physical fitness along the aging process [30, 31].

Finally, leisure time/commuting PA was associated with PA intensities from MVPA An important result of our study is that 20% of this sample reported walking and cycling to work, which would contribute to these findings. Yang et al. [32] observed that adopting active commuting was associated with additional minutes of MVPA per day, mainly among women. This is an important finding since Kwaśniewska et al. [33] observed that participants who walked or cycled had a lower prevalence of cardiometabolic risks. The present study observed different associations between leisure time/commuting and PA intensity according to sex. Women showed a significant association of leisure time/commuting scores with light- and moderate-intensity PA, while men showed an association with vigorous-intensity PA. The absolute sex difference in physical fitness [27–29] could also be related to this domain, as discussed regarding sports practice since male participants can opt to perform leisure activities with higher intensity and walk or cycle at a greater intensity than women. Otherwise, a systematic review by Pollart and Wagnild [34] reported that more women walk for leisure, but this proportion can reverse in older participants. Patterson et al. [35] reported significant gender differences for active commuting, with a more supportive physical environment being associated with more active commuting among men and a stronger association of social environment with active commuting among women. However, the present study was unable to split the leisure time and commuting due to the characteristics of the Baecke questionnaire, which provides a unique score for this domain, being not possible to infer about walking or cycling for leisure or commuting purposes.

Considering the vigorous intensity, only sports practice and leisure time/commuting PA were associated. This finding could be related to the fact that a large part of sports practice comprises vigorous activities, such as resistance training, running, and recreational sports, mostly consisting of intermittent and high-intensity PA [36]. Regarding leisure time/commuting PA, this study observed that the average amount of vigorous-intensity PA was approximately 60% higher in those who walked or cycled to work than those who traveled by car, motorcycle, or bus. When analyzed the very vigorous PA (>8.99 METs or> 9642 counts per minute), only associated with sports practice domain of PA was associated. It was observed that approximately 50% of this study’s participants reported practicing some sport or exercise at leisure, which could explain these findings.

One of the aspects to consider is that the present study was conducted in a city of approximately 20 thousand inhabitants, which can be considered a small city. The types of PA, especially considering the time spent commuting and the distances between home and work compared to inhabitants of large cities, certainly tend to be different, and comparisons in this sense must be considered. In addition, the limited sample size precluded inferences of sensitivity analysis according to sex regarding vigorous and very-vigorous physical activity, presenting a very low average weekly minutes and wide variability in the sample.

As a limitation of the present study, the cross-sectional design of this observational study does not allow for the analysis of cause-and-effect relationships. In addition, it was not possible to stratify the physical activity assessed by accelerometry in the different domains of practice, which needs to be investigated in future studies. As positive aspects, the randomized sampling process and the novelty analysis between PA’s different domains and intensities adjusted for confounding factors.

As practical applications of the present study, we emphasize that health promotion actions prioritizing mild intensities include investing in occupational or commuting PA. In general, sports practice is the type of PA most related to higher intensities (MVPA). It could be an important component of programs to increase physical fitness and cardiovascular and bone health, among other health parameters. However, in populations at higher risk, such as older adults with co-morbidities (such as heart failure or ischemic heart disease), higher intensities of PA should be avoided.

## Conclusion

Based on this study’s findings, PA performed in domains of work, sports practice, and leisure time/commuting was associated with moderate-vigorous intensities, while only sports practice and leisure time/commuting were associated with vigorous-intensity PA. Very vigorous-intensity PA was associated only with the score of PA at sports practice. As practical applications, health promotion actions should provide incentives and conditions for the active commuting of the population to their jobs, as well as focusing on sports practice motivation, aiming to increase the weekly amount of higher intensity PA.
